# Statistical Analysis and Decoding of Neural Activity in the Rodent Geniculate Ganglion Using a Metric-Based Inference System

**DOI:** 10.1371/journal.pone.0065439

**Published:** 2013-05-30

**Authors:** Wei Wu, Thomas G. Mast, Christopher Ziembko, Joseph M. Breza, Robert J. Contreras

**Affiliations:** 1 Program in Neuroscience, Florida State University, Tallahassee, Florida, United States of America; 2 Departments of Statistics, Florida State University, Tallahassee, Florida, United States of America; 3 Department of Mathematics, Florida State University, Tallahassee, Florida, United States of America; 4 Department of Psychology, Florida State University, Tallahassee, Florida, United States of America; Tokai University, Japan

## Abstract

We analyzed the spike discharge patterns of two types of neurons in the rodent peripheral gustatory system, Na specialists (NS) and acid generalists (AG) to lingual stimulation with NaCl, acetic acid, and mixtures of the two stimuli. Previous computational investigations found that both spike rate and spike timing contribute to taste quality coding. These studies used commonly accepted computational methods, but they do not provide a *consistent* statistical evaluation of spike trains. In this paper, we adopted a new computational framework that treated each spike train as an individual data point for computing summary statistics such as *mean* and *variance* in the spike train space. We found that these statistical summaries properly characterized the firing patterns (e. g. template and variability) and quantified the differences between NS and AG neurons. The same framework was also used to assess the discrimination performance of NS and AG neurons and to remove spontaneous background activity or “noise” from the spike train responses. The results indicated that the new metric system provided the desired decoding performance and noise-removal improved stimulus classification accuracy, especially of neurons with high spontaneous rates. In summary, this new method naturally conducts statistical analysis and neural decoding under one *consistent* framework, and the results demonstrated that individual peripheral-gustatory neurons generate a unique and reliable firing pattern during sensory stimulation and that this pattern can be reliably decoded.

## Introduction

Peripheral receptors and their communicating nerves are the information highways linking environmental stimuli with the brain. Sensory perception, and all subsequent behavior, requires the central nervous system to properly decode peripheral activity. In the taste system, cranial nerves (VII, IX and X) connect the oral cavity and the brainstem, providing the necessary input for the human perceptual qualities of: salty, sweet, sour, bitter, and umami. Therefore, taste identification is dependent upon the spike train information transmitted by the neurons from these three cranial nerves.

Of the four nerve branches that innervate taste bud receptor cells in the oral cavity, the chorda tympani (CT) nerve, which innervates the fungiform taste cells in the anterior tongue, may be the most critical for encoding salt taste information in mammals. In rodents, two well-defined neuron types with unique chemical sensitivities (i.e. salty, sour, etc.) and behavioral roles dominate the CT nerve responses to salt. The first salt-sensitive type, Na-specialist (NS) neurons, responds strongly to solutions containing the sodium cation, but not to non-sodium salt solutions or to the other basic taste stimuli [1]. Narrowly-tuned NS neurons are sodium selective because they receive input from taste bud receptor cells expressing the sodium-selective epithelial sodium channel (ENaC) [2]–[7]. In contrast, the second salt-sensitive type, acid-generalist (AG) neurons, are broadly-tuned responding, as the name implies, especially well to acids, but also strongly to most cationic salts like NaCl, KCl, CaCl_2_, and NH_4_Cl [8]–[11]. The sodium detection mechanism for AG neurons is independent from ENaC, but otherwise controversial [12]–[13]. Behaviorally, NS neurons are required for Na^+^-K^+^ discrimination [4] and for the detection of low-concentration Na^+^ [14], [15]. On the other hand, behavioral evidence suggests that AG neurons are used to detect several cations [16], [17]. While AG and NS neurons use different molecular mechanisms, and have different functional roles, their different coding strategies are just beginning to be elucidated.

As discovered by pioneering taste neurophysiologists [18]–[22], peripheral-taste neurons use both spike rate and spike timing to encode chemical stimuli. Both salt-sensitive neuron types increase their firing rates to increases in Na^+^ concentration, but the temporal dynamics differ. NS neurons respond with a short-latency, high frequency phasic response followed by a declining static phase [23], [10], [11]. In contrast, AG neurons respond more sluggishly with a longer latency and a slower phasic response followed by a more sustained static phase [23], [10], [11]. Although NS neurons are unresponsive to a broad range of acetic acid concentrations, acetic acid reduces the firing rate when presented together in a mixture with NaCl [10]. In contrast, AG neurons increase their firing rate to increasing concentrations of both NaCl and acetic acid; when presented together in a mixture, the response rate is additive [10].

Computational analyses of spike timing indicate that response latency plays a significant role in stimulus encoding in the periphery [24], similar to somatosensory touch coding [25]. Accordingly, taste processing in the downstream nucleus of the solitary tract (NST) [26]–[29] and gustatory cortex [30] utilizes temporal information. It has been proposed that a role of the NST is to enhance and propagate the temporal differences in peripheral activity [28] and that forebrain neurons may use this pattern to identify and evaluate Na^+^ taste stimuli [31].

However, without completely taking into account the location of each spike within, the stimulus-evoked spike train, the spike-template pattern cannot be fully defined. This assumes and requires that each stimulus generate a consistent and unique spike template pattern that encodes information about stimulus quality. Each spike template is defined by a *mean spike rate* and a *mean temporally arranged spike pattern* replete with *deviations* from the mean for both values. Spike templates would then be used to assess, for example, how NS and AG neurons encode information. This approach opens several possibilities. For example, is the spike template pattern to NaCl the same or different for NS and AG neurons? When two stimuli are presented together in a mixture like NaCl and acetic acid, how does the spike template pattern change? Does the mixture template reflect the pattern of the dominant stimulus in the mixture or does it reflect the template features of both stimuli? Do NS and AG neurons differ in their spike template patterns to mixtures? These are the kinds of questions raised in the present study.

Spike timing is a component of stimulus decoding, as variation in rate appears to mask central taste coding of NaCl [29]. To our knowledge, a spike template pattern to NaCl has yet to be described in either peripheral or central gustatory neurons. To generate an accurate spike-template pattern requires a metric that considers both the number and temporal distribution of spikes in the stimulus-evoked spike train and then, based on a number of stimulus trials, statistically computes the *mean spike train* and its *associated variance*, which characterizes the deviation from the template. Spike-train metrics, which measure dissimilarity between spike trains, have been widely used to study neural coding. As measures of dissimilarities, many of the past spike train distances have found success in specific scenarios [32–43). The applications of these methods have mainly focused on the clustering or classification of spike trains with respect to different stimuli. Their relevance in deriving statistical summaries (e.g. mean spike train and spike train variance) remains unclear, as the statistics defined with these distances may not provide interesting intuition or tractable computations.

Wu and Srivastava [44] have proposed a novel metric to measure distance between spike trains. This metric corresponds to the conventional Euclidean distance in the function space. It is a proper distance that quantifies dissimilarity between spike trains. Importantly, it can be used to define basic summary statistics, such as sample mean and sample variance, for any set of spike trains providing a consistent framework for spike train analysis. In this study, we adopted this metric as well as the associated summary statistics in spike train space to describe neuronal activity. In particular, we examined typical-firing pattern and spike-train variability across the time domain. Further, we evaluated the similarity of these activities to different stimuli and in the process describe what information these classes transmit. Spontaneous activity has been reported to inflate neuronal breadth of tuning and to obscure inhibitory responses [45]. Consequently, spontaneous or background rate is treated as noise and is simply subtracted from the stimulus evoked response [28], [29], [46], while making inhibitory response more difficult to analyze [45]. The new metric was also used to match spontaneous activity with stimulus-evoked activity to delete noise-related spikes from the spike train responses to chemical stimulation.

## Materials and Methods

### Single-cell recording techniques

#### Geniculate ganglion

Previously recorded neurons were reanalyzed and the methods used to access, stimulate and record these rat geniculate ganglion neurons have been previously reported [10] and approved by the Florida State University Institutional Animal Care and Use Committee. Briefly, adult male Sprague-Dawley rats were anesthetized with urethane (1.7 g/kg body wt), and geniculate ganglion cell bodies were exposed by the dorsal approach [10], [11], [46]–[49].

Solutions were flowed over the tongue at 50 µl/s and 35°C (OctaFlow; ALA Scientific Instruments, Farmingdale, NY). We tested each single neuron's response to 5 s of stimulation with the following stimuli: 0.1 M NaCl, 0.01 M citric acid (CA), 0.003, 0.01, 0.03, and 0.1 M acetic acid (AA), and each acetic acid mixed with 0.1 M NaCl. Each stimulus was presented 2–4 times. Stimulus trials were divided into three regions: a 5-s pre-stimulus period, a 5-s stimulus application period, and a 5-s post-stimulus period.

Activity was recorded extracellularly (criteria 3:1 signal-to-noise ratio) with low-impedance (1.1–1.9 MΩ) glass-insulated tungsten microelectrodes. Off-line, single-units were separated with spike templates based on amplitude and waveform shape (Spike2; Cambridge Electronic Design, Cambridge UK). Cells were identified as *Na^+^ specialists* (Na-S or NS; N = 13) or *acid generalists* (AG; N = 8) based on cluster analysis [Pearson's product-moment correlation coefficient (r–1) and the average-linking method between subjects (Statistica, StatSoft, Tulsa, OK)] of spiking responses to the standard stimuli [11]. These 21 cells were sorted in chronological order, with Cells 1, 2, 4, 5, 7, 8, 12, 14, 16, 17, 18, 19, 20 in the NS group, and Cells 3, 6, 9, 10, 11, 13, 15, 21 in the AG group.

### Statistics Methods

In this section, we present the spike train metric and associated summary statistics such as mean and variance that we used to analyze the spike train responses of geniculate ganglion neurons to chemical stimulation. This metric, *d*_2_, taken from Wu and Srivastava [44] corresponds to the classical “Euclidean distance” between two spike trains. This metric is also closely related to the commonly used

Victor-Purpura [50] and van Rossum metrics [35].

#### “Euclidean” Metric *d*_2_

Assume 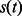 is a spike train with spike times 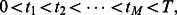 where [0, *T*] denotes the recording time domain. That is, 
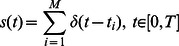



where *δ*(•) is the Dirac delta function. Heuristically, the metric between two spike trains *f* and *g* is computed by composing *g* with a time warping function to match *f*, and the distance is a combination of how closely this warped *g* matches *f* and how much warping was required to achieve this matching.

Let Γ be the set of all time warping functions, where a time warping is defined as a continuous, strictly increasing, and piecewise linear fucntion in the domain [0, *T*]. For 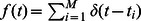 and 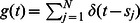, the distance *d*_2_ is defined in the following form: 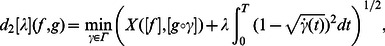
(1)


where λ>0, and *X*(., .) denotes the cardinality of the Exclusive OR of two sets. That is, 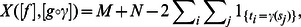



where 1_{.}_ is the indicator function.

The integration in Eqn. 1 includes two terms. The first term, 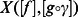, is called matching term which measures the goodness-of-match between *f* and *g* in presence of warping. The second term, 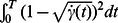 is called penalty term, which penalizes the amount of warping. λ (>0) is the penalty coefficient, which controls the degree of penalty on warping. We emphasize that *d*_2_ is a proper metric; i.e. it satisfies *positive-definiteness*, *symmetry*, and *triangle-inequality*. Indeed, it shares a lot of similarities to the classical *L* ^2^ Euclidean norm in functional analysis, and be further used to define summary statistics such as mean and variance in the spike trian space.

#### Matching algorithms for *d*_2_

To evaluate *d*_2_, we need an optimal time warping that minimizes the penalized warping distance. We used the renowened dynamic programming algorithm to solve for the optimal time wapring, with a finite grid being the spike times of the two spike trains. For two spike trains *f* and *g* that have *m* and *n* spikes respectively, the computational cost will be *O*(*mn*).

#### Summary Statistics

We use the metric *d*_2_ to compute summary statistics in the spike train space. The challenge lies in computing the mean of a sample of spike trains. Once the mean spike train is known, the variance computation follows naturally.

#### Mean and Variance

We defined the sample mean of a set of spike trains using the Karcher mean with the *d*_2_ metric [44]. Given a set of spike trains *S*_1_, *S*_2_, …, *S_N_*, their sample *mean S* ^*^ is defined as follows:
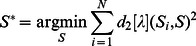
(2)


Given the mean train *S* ^*^, we computed the sample *variance σ* ^2^ (*σ* is the *standard deviation*) in the standard way:
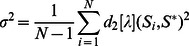
(3)


A useful feature of summary statistics in this manner is that we can partition the domain [0, *T*] to multiple sub-intervals, and then compute the mean and variance in each one of them. The result indicates the trend of the template and variability across the time.

#### Computational Methods

Computation of mean spike train, in general, is a challenging problem. When λ is sufficiently small, there is little penalty on time warping. The framework becomes fully elastic in the time domain and this leads to a more intuitive distance. An efficient and convergent procedure, Matching-Minimization (MM) Algorithm, was constructed to compute the mean in this case [44]. For arbitrary value of λ (> 0), we need to update the number of spikes as well as their locations during the computation. Another efficient procedure, Matching-Centering-Pruning (MCP) Algorithm, was recently developed which generalizes MM-algorithm and also result in convergent estimation [51].

### “Background Noise” Removal

The goal is to understand taste coding by geniculate ganglion neurons. However, these neurons are spontaneously active without stimulation. With stimulation, the spike trains are actually a mixture of background activity and responses to taste stimuli. In this study, we viewed spontaneous activity as “background noise”. The amount of noise varied widely among the neurons. Some neurons exhibited almost no spontaneous activity, while others were quite active.

To focus on the spike activity during chemical stimulation, we aimed to develop a “noise removal” procedure. One naive idea is to compute the average spontaneous firing rate, and then subtract it from the corresponding number of spikes during stimulation. However, the key problem with this approach is that it is arbitrary to assume the information is encoded in the total spike count. The method will fail if spike timing is an important factor in coding. Deletion of spikes based solely on the number of spontaneous spikes may distort the temporal code during stimulation.

We therefore used the *d*_2_ metric to compute the mean spike train during the pre-stimulus period when spontaneous spikes are the only source of activity. This was done separately for each neuron because each one has its own spontaneous pattern. The mean is used as the template for the pre-stimulus time period. For each neuron, we found the optimal match between the template during the pre-stimulus period with each spike train observation during stimulation for all stimuli. We treated any co-incident spikes as noise and therefore removed them from the observed train. This matching process is well described in Wu and Srivastava [44]. Two examples are shown in Figure 6 to illustrate this noise-removal process. The method can be described in the following steps:

1. Computed the mean spike train, *f*, of all spike trains during the 5-s pre-stimulus period.
2. For each train, *g*, during the 5-s period of stimulation, we computed the spike train distance between *f* and *g*, and found the optimal warping *γ* in *g*.
3. Removed all spikes in *g* that matched (via *γ*) with spikes in *f.*

## Results

In this section, we applied the spike train metric *d*_2_ as well as the associated summary statistics (mean and variance) to perform analysis on the recorded geniculate ganglion data. We also used these tools to decode neural activity and classify the taste stimuli. We first performed some basic analyses to understand the typical firing pattern and the associated variability.

### Data and the Distance Measurement

**Figure 1:**
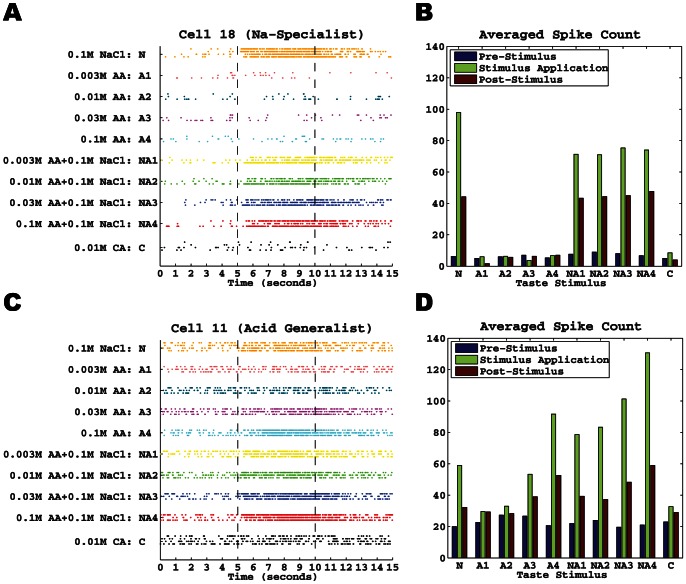
Typical response patterns in each group. **A.** Raster plot of all spike trains of a Na-Specialist (Cell 18) with respect to 10 different stimuli. Each trial was divided into three regions: a 5-s pre-stimulus period, a 5-s stimulus application period, and a 5-s post-stimulus period. Dots on each row denote the spike times. For simplification, we use letters N, A1, A2, A3, A4, NA1, NA2, NA3, NA4, C, to denote the 10 taste stimuli, respectively. **B.** Averaged numbers of spikes of each stimulus in the pre-stimulus period (blue bars), stimulus application period (green bars), and post-stimulus period (red bars), respectively. **C and D.** Same as A and B except for an Acid Generalist (Cell 11).

As stated in the Methods Section, we assessed the responses of 21 geniculate ganglion neurons (13 NS & 8 AG) with respect to 10 different taste stimuli. Figure 1 shows the typical response patterns from one neuron in each group. The NS neuron (Cell 18; Fig. 1A) had a low spontaneous rate during the 5-s pre-stimulus period. This neuron responded significantly to NaCl and mixtures of NaCl with acetic acid, but very little to acetic acid or citric acid during 5-s stimulation period. During the 5-s post-stimulus period, the firing rate subsided gradually approaching the pre-stimulus rate at the end of the period.

Figure 1B shows the average spike count to the 10 stimuli for this cell during each 5-s time period. As can be seen, the pre-stimulus spike count was similar for all 10 stimuli. This NS neuron responded robustly to NaCl (∼20 spikes/sec), while the responses to acetic acid and citric acid were similar to spontaneous firing rate. The responses to the NaCl + acetic acid mixtures were lower than that to NaCl alone, but greater than that to acetic acid alone.

Figure 1C and 1D shows the typical responses of an AG neuron (Cell 11). In contrast to the NS cell, this AG cell responded well to all stimuli. The firing rate increased with increasing stimulus concentration to acetic acid alone as well as to the NaCl + acetic acid mixture. Moreover, the firing rate to each mixture was higher than that to either NaCl or acetic acid alone. This additive pattern for the AG neuron is opposite to the suppressive pattern seen for the NS neuron.

**Figure 2:**
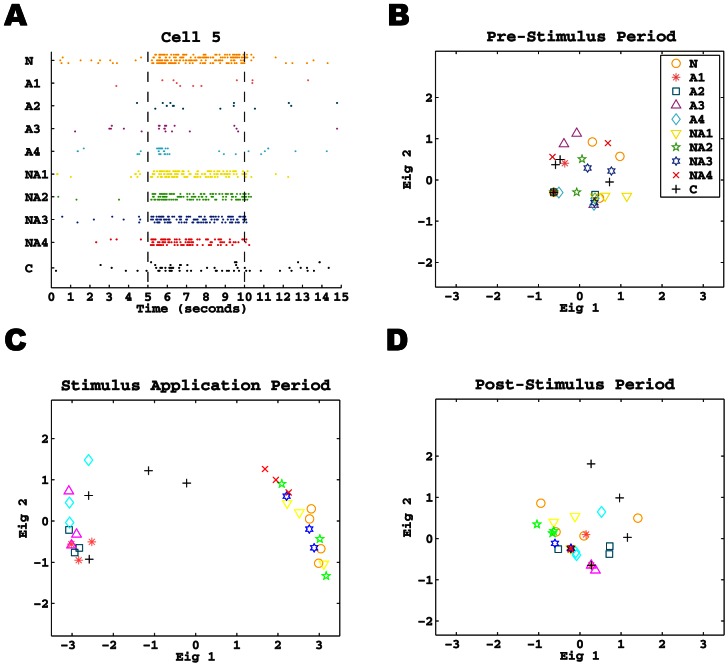
Illustration of the *d*_2_ metric with one example neuron. **A.** Raster plot of all 32 spike trains of a Na-Specialist (Cell 5) with respect to the 10 different stimuli. The data is plotted in the same form as that in Fig. 1A. **B.** Scatter plot of the first two components in the Multidimensional Scaling (MDS) during the pre-stimulus period. The trains under each stimulus are denoted by the same symbol (see legend), and the colors on the symbols are consistent to that in A. **C and D.** Same as B except for the stimulus application period and post-stimulus period, respectively. These two plots are in the same scale as that in B for comparison on variability.

Next we adopted the “Euclidean” metric *d*_2_ (see Methods) to measure the dissimilarity between two spike trains [44], [51], and allowing comparison of both firing rate and pattern between different spike trains within a single metric. The appropriateness of this metric is demonstrated using one example neuron (Cell 5) in Figure 2. The firing activity of this NS cell is shown as a raster plot in Figure 2A. The pairwise distances for all 32 spike trains (for 10 stimuli) in pre-stimulus period (first 5-s) were then computed. A classical multidimensional scaling (MDS) analysis was conducted using these distances, and a scatterplot with the two most prominent components is shown in Figure 2B. In this plot, each spike train is represented as a two-dimensional point, and the locations of all points characterize their relative distances. As the distances are computed in the pre-stimulus period (spontaneous activity), Figure 2B shows that there was no clear separation among 10 stimuli. That is, all points are closely distributed in one cluster demonstrating that this NS cell had stable spontaneous rate for the ∼50-min period of the recording. In contrast to the pre-stimulus case, the points in the MDS plot generated from the stimulus application period (Fig. 2C) are more dispersed, due to relatively larger stimulus-dependent distances. In general, the points separate largely into two main clusters: NaCl and NaCl + acetic acid mixtures are located in close proximity to each other, but separate from acetic acid and citric acid which are located together in another broader cluster. In the post-stimulus period, the spike activity returned to baseline and the MDS plot forms a single cluster (Fig. 2D) much like the pre-stimulus plot (Fig. 2B). In summary, this illustrates the power of metric *d*_2_ to separate spike trains into different clusters based on their responses to chemical stimuli; similar spike trains have short distances, and dissimilar ones have large distances.

### Estimating Spike Train Templates

**Figure 3:**
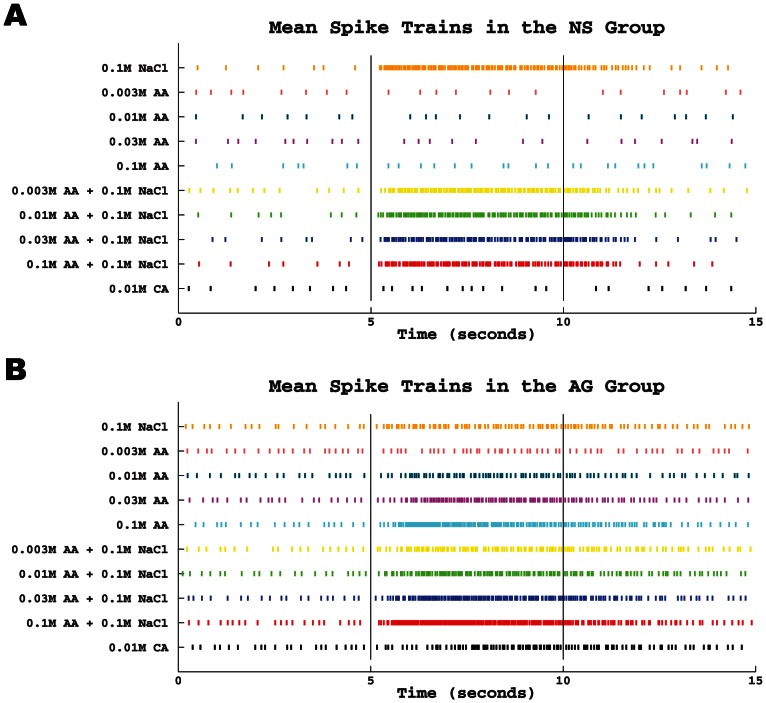
The mean spike train for each neuron group. **A.** Each row is the mean spike train of all trains in the NS group (for all 13 neurons) for a specific taste (shown along the y-axis). Short, vertical lines on each row denote the spike times and the colors are consistent to that in Fig. 2. **B.** Same as A except for the AG group (for all 8 neurons).

We computed the mean spike train or template pattern for each taste stimulus. Separate means were computed for each of the three time intervals to characterize the templates before, during, and after stimulus application. The resulting means are concatenated and shown in Figure 3A for NS neurons. During the pre-stimulus period the mean spike trains were similar for the 10 stimuli indicating stable spontaneous activity. In addition, the mean spike trains to the four concentrations of acetic acid and the single concentration of citric acid appeared similar to that during the pre-stimulus period. In contrast, NS cells responded to NaCl and the NaCl + acetic acid mixtures with a short latency burst of activity that was sustained at a progressively slower rate for the 5-s stimulation period. However, the template pattern to the mixtures was more tempered in immediate firing rate and had more brief pauses in spiking during the 5-s stimulation period compared to the template pattern to NaCl. For example, if a pause is defined as an inter-spike interval with duration of at least 75ms, we found that the number of pauses to the NaCl stimulus was 10, but that to the four mixtures were 12, 12, 16, and 16, respectively. During the post-stimulus period, the spike discharges to NaCl and the mixtures subsided gradually. This gradual decline to baseline level occurred more quickly to the mixtures and increased progressively with acetic acid concentration. Thus, even though NS neurons were largely unresponsive to acetic acid, acetic acid, nevertheless, altered the template pattern to NaCl during the stimulus and post-stimulus periods.

Figure 3B shows the mean spike trains of AG neurons for the three periods. Like for NS neurons, the mean spike trains for AG neurons were similar for the 10 stimuli during the pre-stimulus period, although at a higher overall baseline rate (compare Fig. 1A and Fig. 1B). During the stimulus application period, AG neurons responded to all stimuli, albeit at different rate and temporal pattern. AG neurons responded to NaCl not with an immediate burst of activity but with a sustained level throughout the 5-s stimulus application period. In response to acetic acid, the mean spike train increased in rate and decreased in latency as concentration increased. The additive effect of NaCl mixed with acetic acid was a further increase in spike rate and a decrease in response latency. During the post-stimulus period, AG neurons were distinguished by the gradual recovery to baseline that lengthened in duration as acetic acid concentration increased, but seem to shorten in duration when NaCl was mixed with acetic acid. In summary, the mean spike trains distinguished NS from AG neurons during all three stimulus periods.

### Measurement of Spiking Variability across Time Domain

**Figure 4:**
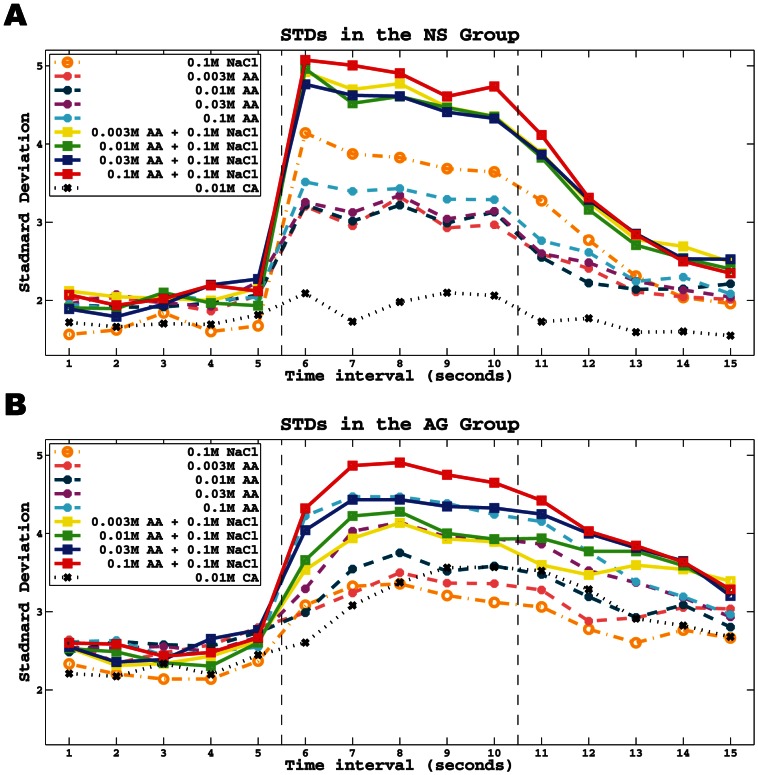
The average standard deviation during the pre-stimulus, stimulus and post-stimulus periods. **A.** Each colored line is the standard deviation (STD) of spike trains in every 1-s period (shown in x-axis) in the NS group for a specific taste (shown in the legend). The colors are consistent to that in Fig. 3. **B.** Same as A except for the AG group.

We also computed the spike train standard deviation (STD) in 1-s intervals across the three stimulus periods according to Eqn. 3 (in Materials and Methods). The results of this analysis are shown in Figure 4A and 4B for NS and AG neurons, respectively. Overall, the variation was consistently low during the pre-stimulus period, sharply increased in the first second of the stimulus application period, and then gradually recovered to baseline in the post-stimulus period. This is consistent with the mean spike trains in Figure 3 because higher firing rates often result in larger distances from the mean (illustrated in Fig. 2C). This is evident during the pre-stimulus period where the STD of the AG group was greater than that of the NS group most likely due to the higher baseline rates of AG neurons (∼ 4 spikes/s for AG; ∼1.5 spikes/s for NS).

During the stimulus application period, there are several important features of the response variability in NS neurons to note. Firstly, although NS neurons were largely unresponsive to acetic acid in terms of response rate (See Fig. 3A), they were clearly affected by this stimulus as indicated by a significant increase in STD during stimulation, which was similar for the four concentrations of acetic acid. Interestingly, 0.01 M citric acid had minimal effect on STD while equimolar acetic acid had a large effect. Furthermore, the response variability was highest to NaCl + acetic mixture than to NaCl alone even though the response rate was greater to NaCl alone than the mixture. In contrast to NS neurons, the STD pattern of AG neurons consistently matched response rate. The response variability gradually changed along a concentration or intensity continuum (Fig. 4B) that was greater to the mixture than to acetic acid alone. Unlike NS neurons, citric acid increased the STD in AG neurons, although perhaps to a slightly lesser degree than to equimolar acetic acid. Finally, NS and AG neurons differed in the time of peak response variability during stimulation. NS neurons peaked early during the first second, while AG neurons peaked later during the stimulation period. The general patterns noted above during the stimulus application period were carried over to the post-stimulus period for both NS and AG neurons.

### Neural Classification of Taste Stimulus

In this subsection, we used the same metric to assess the classification accuracy of NS and AG neurons.

### Decoding by stimulus and by category

It is important to understand how the peripheral nervous system uses spiking activity to classify different chemical stimuli for taste. Consequently, we analyzed the classification accuracy for each of the 21 neurons relying solely on the spiking activity during the 5-s period of stimulation.

**Figure 5:**
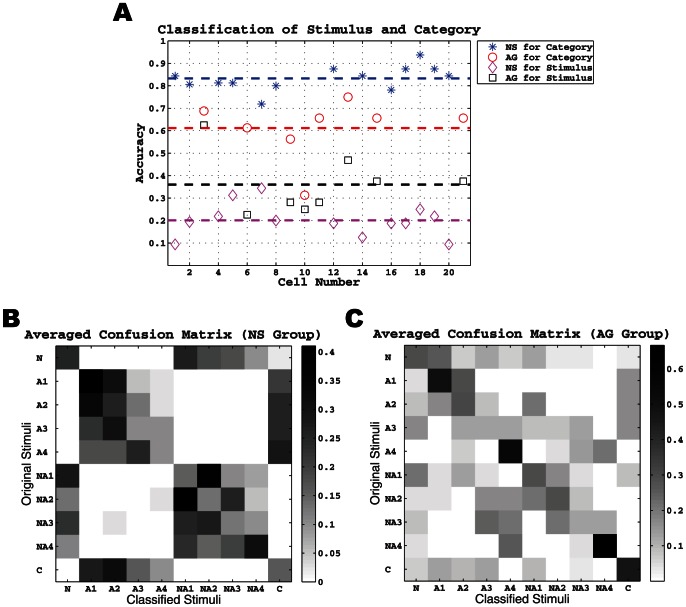
Difference in classification accuracy by cell number and confusion matrix by neuron group. **A.** Classification result over all 21 neurons. Blue stars and red circle denote the classification accuracy on taste categories (i.e. NaCl, acids, or mixtures) in the NS and AG groups, respectively. Black squares and magenta diamonds denote the classification accuracy on individual taste stimuli in the two groups, respectively. Each dashed line denotes the averaged accuracy in the corresponding group where the color matches. **B.** The average confusion matrix in the NS group. Each (*i*, *j*) entry in this matrix corresponds to the averaged (over all NS neurons) probability that the *i*-th stimulus is classified to the *j*-th one. **C.** The average confusion matrix in the AG group.

We first determined whether neurons responded with a unique spike train to each of the 10 stimuli. Due to the low number of stimulus repetitions (2–4), we adopted a standard leave-one-out cross-validation procedure to measure classification accuracy (Lawhern et al, 2011). The results of our analysis are shown in Figure 5A for all 21 neurons. The average accuracy for the NS neurons was only about 20%. Although low, it is still twice as high as a random guess (10%). In contrast, AG neurons had an average classification accuracy nearly twice that of the NS neurons (37%) and four-times greater than chance.

**Figure 6:**
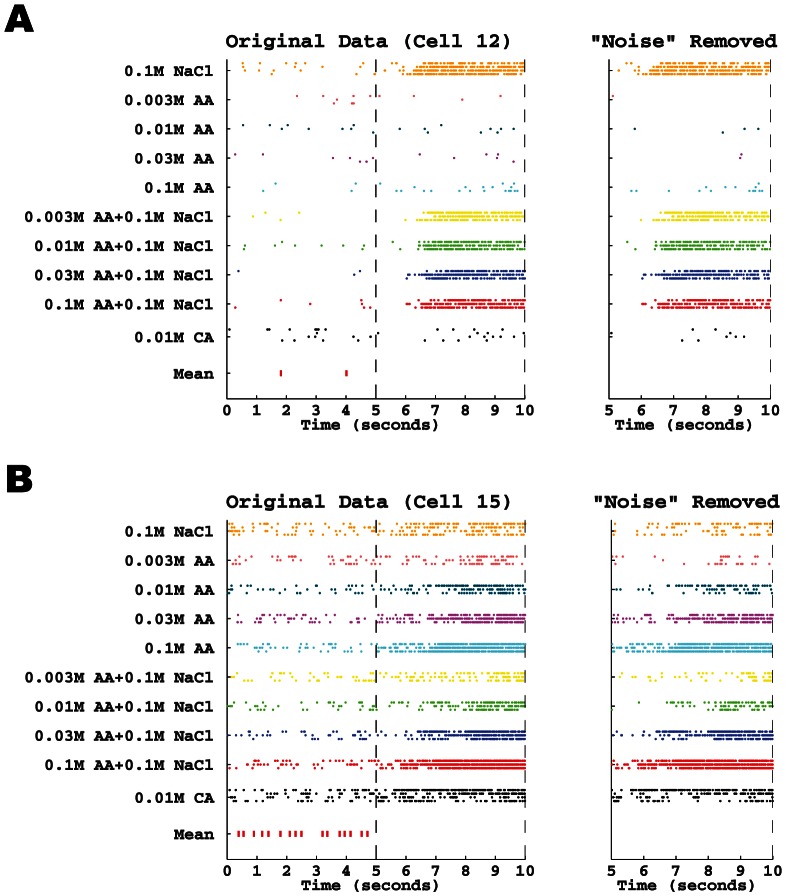
Raster plot of original spike train data and plot of same data with “noise” removed. **A.** Left panel: Spike trains of a Na-Specialist (Cell 12) with respect to the 10 stimuli in the pre-stimulus and stimulus application period. Dots on each row denote the spike times. The mean spike train in the pre-stimulus period is also shown using vertical lines in the bottom row in the data. Right panel: The “noise-removed” spike trains during the stimulus application period. **B.** Same as A except for an Acid Generalist (Cell 15).

The rate and mean spike train analysis depicted in Figures 1–3 showed that NS neurons were more responsive primarily to qualitative differences in the stimulus set and less sensitive to intensity differences, while AG neuron were more responsive to intensity differences. Therefore, we next determined whether classification accuracy improved by analyzing stimulus categories. The 10 stimuli were separated into 3 different categories: NaCl only, 5 acids (4 acetic acid concentrations and 1 citric acid), and 4 NaCl + acetic acid mixtures. The same leave-one-out cross-validation procedure was used to measure the classification accuracy for stimulus category (see Fig. 5A). Analyzing by category significantly improved the classification accuracy to 82% for NS neurons and 60% for AG neurons.

To examine the discrimination performance of geniculate ganglion neurons, we performed a cluster algorithm of each neuron's responses to the 10 stimuli. Figure 5B shows the averaged clustering or confusion matrix for all NS neurons. Here we first computed the probability of classifying stimulus *i* (the rows of the matrix) to stimulus *j* (the columns of the matrix) for each NS neuron [24]. Each (*i*, *j*) entry in this matrix then corresponds to the average of these probabilities. It was found that NS neurons segregate mostly within quadrants of the matrix. This segregation reflects the fact that acetic acid trials were classified as one of the four acetic acid concentrations and NaCl + acetic acid trials were classified as one of the four mixture concentrations, but acetic acid was nearly never classified as or confused with NaCl + acetic acid and vice versa. On some trials, NS neurons confused acetic acid with citric acid, and NaCl with NaCl + acetic acid. This matrix illustrates how NS neurons obtained a classification accuracy of 82% for stimulus category. In contrast to this pattern for NS neurons, the matrix pattern for AG neurons (see Fig. 5C) was quite different where several taste stimuli can be classified with high accuracy (large probabilities along the main diagonal). This reflects the fact that AG neurons were responsive to differences in stimulus intensity. The matrix also shows that AG neurons occasionally confused acetic acid with NaCl + acetic acid. This confusion primarily accounts for the 60% performance by AG neurons in classification accuracy for stimulus category.

### Decoding on Spike Trains with “Background Noise” Removed

Figure 1 shows that NS and AG neurons have different spontaneous activity patterns. To enhance our analysis of sensory coding, we adopted a new procedure that removes background noise from the response record based on the rate as well as the temporal pattern of spontaneous activity. Figure 6 shows an example of how noise removal enhanced the NaCl selectivity of some NS neurons and the responsiveness of AG neurons to differences in stimulus concentration.

**Figure 7:**
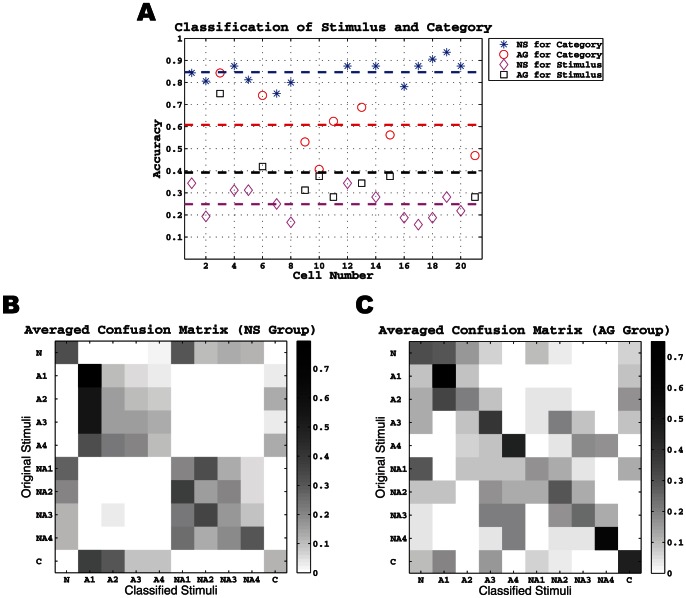
Classification accuracy and the confusion matrices in NS and AG groups using “noise-removed” spike trains. The plot in **A** and two images in **B** and **C** are the same as those in Fig. 5 except that the background firing activity has been removed.

After removing the noise from all the neurons' responses, we re-evaluated classification accuracy separately for NS and AG neurons first for the 10 stimuli and also by the three stimulus categories (NaCl, acids, NaCl + acetic acid mixtures). The results of this analysis are plotted individually for each neuron in Figure 7A. We found that noise removal improved classification accuracy only by a small margin. The average classification accuracy for the stimuli increased from 20% without noise removal to 25% with noise removal in NS neurons, while that for AG neurons increased from 36 to 40%. The classification accuracy for stimulus category, however, remained largely unchanged at 82% for NS neurons and 60% for AG neurons. We also computed the average confusion matrices for NS and AG neurons after noise removal, and the result is shown in Figure 7B, C. As can be seen, there was little change in the matrix profiles. The NS matrix largely retained its quadrant-like appearance, while the AG matrix concentrated its activity along the diagonal. In sum, noise removal had only a modest effect in improving the classification accuracy of geniculate ganglion responses to chemical stimuli. However, noise removal was beneficial for some neurons and not others.

**Figure 8:**
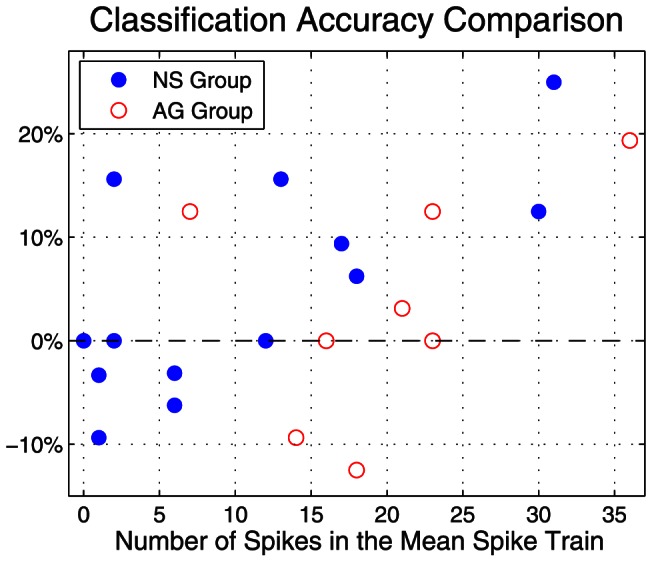
Difference of classification accuracies using the original spike trains and “noise-removed” spike trains with respect to the number of spikes in the mean spike trains in the pre-stimulus period. Blue dot and red circle denote the cells in the NS and AG groups, respectively.

Therefore, we determined whether classification accuracy varied with respect to the number of spikes in the mean spike trains. For each neuron, we computed the classification accuracy to the 10 stimuli using the noise-removed data as well as the accuracy using the original data. It was found that this difference (former minus latter) is approximately an increasing function with respect to the number of spikes in the mean spike trains (see Fig. 8). This was especially clear for NS neurons. This result also shows that when the spontaneous rate was above the average spontaneous rate (median spike count  =  20 for AG neurons; 8 for NS neurons), noise removal improved classification accuracy. In contrast, when spontaneous rate was low, noise removal had no effect or degraded the decoding performance.

## Discussion

The present findings demonstrated that individual geniculate ganglion neurons generated a unique and reliable firing pattern during chemosensory stimulation and that this pattern could be reliably decoded. Narrowly-tuned Na^+^-sensitive (NS) neurons were best at categorical discrimination responding with spike templates that distinguished NaCl, acids, and NaCl + acid mixtures from each other. They were relatively poor at distinguishing differences in acetic acid concentration when presented alone or in a mixture with NaCl. Conversely, broadly-tuned acid-generalist (AG) neurons were best at discriminating differences in stimulus concentration responding with spike templates that distinguished one acetic acid concentration from another and one mixture concentration from another. These results support previous evidence that NS neurons encode stimulus identity while AG neurons encode something akin to stimulus intensity [52]. Lastly, the templates provide strong evidence that spontaneous activity, or noise, occurs within the response epoch and a statistical approach [44] to removing noise can improve discrimination performance for neurons with high spontaneous rate.

Our results provided insight into geniculate ganglion coding of taste information. Given our stimulus set, NS neurons may not simply transmit information about the presence or absence of NaCl. Instead, they may transmit information about NaCl + X, where X is a chemical stimulus such as acetic acid. Indeed, NS neurons generated unique spike templates for each of the three stimuli (NaCl, acids, or mixtures of the two; Fig. 3A). Furthermore, NS neurons never confused NaCl with acetic or citric acid, and only rarely did they confuse NaCl with NaCl + acetic acid (Fig. 5A). This seemed to depend on stimulus concentration. NaCl mixed with weak acetic acid was more often confused with NaCl than when mixed with strong acetic acid. Thus, the resulting code may be that of a weak NaCl concentration when NaCl is mixed with weak acetic acid, and something quite different when mixed with strong acetic acid. Interestingly, humans do not mistake NaCl + acid mixtures for NaCl alone [53], but this may depend on acid concentration like for NS neurons.

Despite the seemingly eponymous name, AG neurons do not transmit identity information about acids. At first glance the confusion matrices and identity graphs appear to support AG neurons ability to identify individual tastes (Fig. 7). However, this could be an artifact of the stimulus battery. AG neurons routinely misclassified chemical stimuli with similar solute concentrations, lumping dissimilar chemicals in the same confusion matrix bin (i.e. high acid is categorized as moderate acid and salt). Instead, AG neurons increased activity along an ionic-concentration continuum and appear to transmit information about stimulus concentration—not stimulus quality. These results agree with previous behavioral studies where animals that lack the NS pathway confuse different salts of the same concentration [4]. Thus, the chorda tympani nerve may use a labeled-line approach with specific tastes (i.e. sodium) and an across-fiber pattern for concentration. This is a possible source of confusion in evidence for “labeled-line” or “across-fiber” taste coding [7], [54] and evidence for more complex taste coding than either coding strategy [9].

The new statistical metric [44] applied to responses by geniculate ganglion neurons also proved advantageous in illuminating differences in response variability between NS and AG neurons (see Figure 4). Perhaps not surprisingly, the differences observed in variability paralleled those observed in mean spike trains or templates. Like mean spike trains, NS neurons segregated mostly into three variability clusters consisting of the NaCl + acid mixtures, NaCl, and the four acetic acid concentrations during the stimulus application and post-stimulus periods. The one notable exception was the unique variability pattern to citric acid, apart from acetic acid, which increased modestly during the stimulus application period and returned quickly to baseline during the post-stimulus period. In contrast to NS neurons, the variability pattern of AG neurons distributed evenly across a stimulus-concentration continuum with the variability pattern to citric acid falling in line with the acetic acid-concentration continuum.

These stimulus-elicited changes and group differences in variability patterns raise intriguing questions. For example, why does acetic acid change the variability of NS neurons when there is no discernible change in response rate? NS neurons were clearly influenced by acetic acid; but is acetic acid a “stimulus” in the traditional sense for NS neurons? This seems unlikely simply because variability was unresponsive to changes in acetic acid concentration. The variability distribution for acetic acid concentration fell within one tight grouping as did the distribution for NaCl + acetic acid concentration. We suspect that processing events occurring in the taste buds may underlie changes in variability, especially in taste receptor cells that express the epithelial sodium channel (ENaC) and communicate with NS neurons. By decreasing intracellular pH, acetic acid inhibits the open probability of the ENaC [54]. By altering the ENaC, acetic acid can increase response variability when added to sodium-containing artificial saliva or when mixed with 0.1 M NaCl. This might suggest that the more acidic citric acid (pH = 2.6) should have a bigger impact on ENaC and increase variability more than acetic acid (pH = 3.55–2.82). However, the opposite occurs because acetic acid is one-third the size of a citric acid, is more lipophilic, and more easily crosses the plasma membrane to decrease intracellular pH [55]. Thus, weak acids like acetic and citric may not be true “stimuli” for NS neurons; however, acidic solutions can change the background conditions for NS neurons and alter their responsiveness to NaCl stimulation. In contrast, weak acids are true stimuli for a separate group of taste bud receptor cells that express PKD channels on their apical membrane [56], [57] and communicate with AG neurons. It is most likely through this acid-sensing mechanism that underlies AG neuron responses to acetic and citric acid along a concentration continuum.

The present results show that the Wu and Srivastava statistical metric [44] is a valuable tool for computing stimulus-evoked spike templates, which may used for gaining insight on how peripheral as well as central neurons encode information about taste quality. Previous work conducted by many investigators have described some of the important signaling properties of peripheral neurons, such as spike rate (e.g. [25]), interspike interval (e.g. [58]), and response latency (e.g. [24]), but until now there hasn't been a statistically sound way to characterize the spike train record in its entirety [44]. Prior studies using electrical brain stimulation mimicking the rate and temporal patterns of sweet sucrose and bitter quinine have obtained mixed success in eliciting specific taste-mediated behaviors. For example, electrical stimulation of the nucleus of the solitary tract (NST) with a spike pattern indicative of quinine stimulation terminated the licking behavior of thirsty rats drinking water [59], [60], and stimulation of the NST in an area associated with quinine taste processing can elicit the aversive reaction of gaping [61]. Additionally, when conditioned to avoid sucrose, some thirsty rats stopped drinking water while receiving brain stimulation that mimicked the pattern for sucrose [62]. While the results of these brain stimulation studies did not always result in the predicted behavioral response, they are valuable for providing evidence that temporal patterns are important for stimulus encoding. We suspect that using the spike templates generated from peripheral geniculate ganglion neurons may improve the success of brain stimulation in eliciting taste-mediated behavior, but this is yet to be determined. Regardless, the ability to generate a mathematically replete mean spike template for each basic taste stimulus and neuron group may improve neuroprosthetics [62].

Peripheral nerve responses contain noise that ordinarily does not degrade the intended encoded signal. For example, NS neurons were able to encode at least three responses: NaCl, not NaCl, and NaCl mixed with something of sufficient concentration. These three responses were evident when no attempt was made to subtract noise (Fig. 5A), when the spontaneous template was carefully matched and subtracted from the response template (Fig 7A) or when the spontaneous rate was simply subtracted from the response rate [10]. However, NS and AG neurons with spontaneous rates above the respective group mean rate appear to transmit ‘noisier’ information. Subtracting the spontaneous template from the response templates in these neurons increased the accuracy of the information transfer (Fig. 8). The source of these ‘extra’ spontaneous spikes is impossible to determine. It is possible that the higher spontaneous rate is due to intrinsic properties of the neurons themselves or to uncontrollable changes in the experimental preparation—anesthesia level, physical impingement of the neuronal membrane by the recording electrode, etc.—and is not a component of the natural system. However, having neurons with the same stimulus-space (i.e. those that respond to the same stimuli) with different spontaneous activity levels have been proposed to enrich sensory coding [63].
